# Space-Time Cluster Analysis of Invasive Meningococcal Disease

**DOI:** 10.3201/eid1009.030992

**Published:** 2004-09

**Authors:** Christian J.P.A. Hoebe, Hester de Melker, Lodewijk Spanjaard, Jacob Dankert, Nico Nagelkerke

**Affiliations:** * Eastern South Limburg Municipal Public Health Service, Heerlen, the Netherlands;; † National Institute of Public Health and the Environment, Bilthoven, the Netherlands;; ‡ Netherlands Reference Laboratory for Bacterial Meningitis, Amsterdam, the Netherlands

**Keywords:** Neisseria meningitidis, invasive meningococcal disease, outbreak, cluster analysis, space-time analysis, secondary cases, nearest-neighbor, research

## Abstract

Clusters are recognized when meningococcal cases of the same phenotypic strain (markers: serogroup, serotype, and subtype) occur in spatial and temporal proximity. The incidence of such clusters was compared to the incidence that would be expected by chance by using space-time nearest-neighbor analysis of 4,887 confirmed invasive meningococcal cases identified in the 9-year surveillance period 1993–2001 in the Netherlands. Clustering beyond chance only occurred among the closest neighboring cases (comparable to secondary cases) and was small (3.1%, 95% confidence interval 2.1%–4.1%).

## Abstract 2

Field clusters are commonly misinterpreted as clusters and would require genotyping to rule out misclassification.

An outbreak of invasive meningococcal disease is a public health emergency because of the disease's unpredictability, sudden lethality, and serious sequelae. Although risk factors are known, the reasons for developing invasive disease are not fully understood. Most persons, when colonized with *Neisseria meningitidis*, become asymptomatic carriers and are sources for further transmission. The apparently sporadic occurrence of invasive disease reflects invisible transmission chains of circulating strains, since invasive disease develops in only a small proportion of those infected. The precise mechanisms generating clusters or outbreaks puzzle public health workers, epidemiologists, and microbiologists (*1**,**2*).

During the 9-year period 1993–2001, the Netherlands had a population between 15.3 and 16 million and encompassed 33,900 km^2^. Most of the ≈500 annual reports of meningococcal disease were sporadic cases, and serogroup B is the most common. From 1993 to 2001, the number of reported cases was from 422 to 770 per year; the peak occurred in 2001 as a result of an increase in serogroup C meningococcal cases. The mean incidence, based on reports of ≈3.4 per 100,000 per year, is comparable to that in England and Wales (3.7) (*3*) but three times higher than in the United States (1.1) (*4*). The Dutch policy for preventing secondary cases compares to the policy in most Western countries and is based on identifying and prophylactically treating close contacts. When two or more possibly related cases (secondary case or cluster) are identified, group contacts in an educational institution (daycare center or primary school) also receive prophylaxis with rifampicin. In the Netherlands, routine vaccination of children for serogroup C meningococcal disease was implemented in September 2002. Furthermore, from June to October 2002, a vaccination campaign was carried out for all 1- to 18-year-olds in response to the increase of serogroup C cases in 2001 and 2002.

Outbreaks are recognized when place (e.g., an educational institution like a primary school), time (e.g., within 1 month), and conventional phenotypic markers (same serogroup, serotype, and subtype) make a connection likely (field cluster) or when an excess of incidence (e.g., 20x normal) is noticed in a retrospectively specified geographic or population area within a chosen period (community outbreak). Field clusters and community outbreaks are rarely seen in the Netherlands, possibly because of underreporting. A group of unrelated cases that occur in temporal and spatial proximity may be misinterpreted as a cluster or outbreak, but these cases would not justify additional public health measures, except perhaps to reassure the public. In a real cluster, cases of the same strain occur in temporal and spatial proximity at a higher frequency than by chance. The objective of our study was to explore the phenomenon of meningococcal clustering in a more objective way by using a nearest-neighbor analysis in space and time that compares the actual occurrence of clusters with their background incidence.

## Patients and Methods

### Data Collection

We used data collected from two surveillance sources: mandatory reports from January 1993 through May 2001 and reports of laboratory-confirmed *N. meningitidis* isolates collected by the Netherlands Reference Laboratory for Bacterial Meningitis in the same period. Additionally, reports of field clusters occurring during the same time were collected as reference.

### Reported Cases

Report data were obtained from the Inspectorate of Health Care. According to the Communicable Disease Act, physicians must report cases of meningococcal disease to their Municipal Public Health Service. The case definition for report includes clinical meningococcal disease in combination with microbiologic confirmation: *N. meningitidis* isolated from blood or cerebrospinal fluid (CSF); meningococcal antigen or DNA detected in cerebrospinal fluid by latex agglutination or polymerase chain reaction; or gram-negative diplococci detected in cerebrospinal fluid, blood, or skin biopsy. The following information was available on an individual level: date of birth, gender, initials, postal code, municipality, date of report, date of first symptoms, date of diagnosis, and age at notification.

### Laboratory Isolates

The reference laboratory collects meningococcal strains from patients with meningitis or septicemia, isolated from blood or CSF. Strains are sent on a voluntary basis to the reference laboratory by all clinical microbiologic laboratories throughout the country. A strain is defined as an isolate of *N. meningitidis* from a patient. When two strains have the same phenotypic markers (serogroup, serotype, and subtype), these are considered to be identical and to belong to one serosubtype. The following information was available for individual patients: date of birth, gender, initials, municipality, date of sample collection, submitting laboratory, date of receipt of strain, date of blood culture, date of lumbar puncture, source of isolate (blood or CSF), serogroup, serotype, and subtype.

### Record Linkage

Records between these two sources were linked (case ascertainment) by using SAS version 8.1 (SAS Institute Inc., Cary, NC). First, records were linked by date of birth, gender, and initials. Records remaining unlinked were then linked by combinations of two variables. The links in the first step were considered correct, while all further links were checked manually for consistency in data fields, spelling mistakes in initials, date of birth, and municipality. In Table 1, we provide an overview of the number of cases and serogroup profile of the data used in our analysis.

**Table 1:** Overview of meningococcal disease cases and serogroup profile of cases included for analysis, 1993–2001

Characteristic	1993	1994	1995	1996	1997	1998	1999^a^	2000	2001^b^	Total
Reported cases (per 100,000 population)	563 (3.7)	422 (2.7)	460 (3.0)	482 (3.1)	491 (3.2)	505 (3.2)	531 (3.4)	516 (3.3)	770 (4.8)	4,740 (3.4)
Case-ascertainment (per 100,000 population)	753 (4.9)	571 (3.7)	689 (4.5)	659 (4.3)	658 (4.2)	704 (4.5)	597 (3.7)	532 (3.4)	396 (5.9)	5,559 (4.3)
Nonconfirmed cases^c^	115	91	88	94	103	86	44	1	41	663
Meningococcal cases included for analysis	638	480	601	565	555	619	553	531	354	4,896
Serogroup profile^d^										
A	3	2	0	0	2	0	0	0	0	7
B (%)	524 (82)	399 (83)	527 (88)	498 (88)	458 (83)	536 (87)	455 (82)	413 (78)	229 (65)	4,039 (82)
C (%)	101 (16)	65 (12)	57 (11)	57 (11)	81 (15)	72 (13)	79 (15)	103 (19)	114 (21)	729 (15)
W135 (%)	4 (1)	5 (1)	7 (1)	3 (1)	6 (1)	4 (1)	12 (2)	12 (2)	7 (2)	60 (1)
X	1	0	0	1	2	1	1	1	0	7
Y	4	5	7	5	6	2	5	2	3	39
Z	0	0	1	0	0	0	0	0	2	3
29E	0	2	0	1	0	1	0	0	0	4
Not serogroupable	1	2	2	0	0	2	1	0	0	8

^a^Case-ascertainment (number of cases after linking procedure) was hampered due to lack of identifying variables from April 1, 1999, when the new Dutch Communicable Disease Act was introduced. ^b^Laboratory data included from January 1993 to May 2001; during the year 2001, the surveillance was more active because of the increase in serogroup C cases. ^c^After linking the reported cases with the laboratory cases, no strain was available for these cases. ^d^Of 4,896 confirmed cases, 9 could not be used in the analysis because of recording errors: 2 serogroup A cases, 4 serogroup B cases, 1 serogroup C, 1 serogroup W135, and 1 serogroup Y.

### Field Cluster

After notification of meningococcal disease, the Municipal Public Health Service considers taking public health measures. Depending on the attentiveness of the communicable disease consultant, field clusters are recognized and reported to the Inspectorate of Health Care, which made this information available for our investigation. Accurate data on actual rifampicin prophylaxis were not available. Field clusters were named after their probable transmission route: family, daycare center, primary school, or swimming pool.

### Statistical Analysis

**Figure 1:**
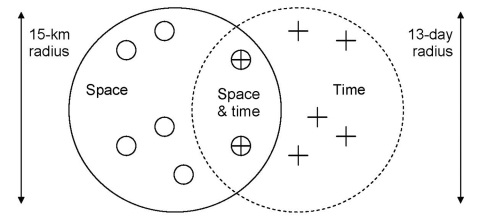
The concept of space-time nearest-neighborship. Nearest-neighbors in space-time are defined as cases that are nearest-neighbors in both space and time. To define the *k*th nearest-neighbors in space-time, we chose the number *n* (e.g., *n* = 7; thus 7 cases [O] occurring within 15 km and 7 cases [+] occurring within 13 days) in each of the neighborhoods so that the number of cases occurring in the intersection of the two neighborhoods (Å) equals exactly *k* (e.g., *k* = 2, the first and second nearest-neighbor in space-time). The radius is shown by the data, given a certain *n*. For a fixed chosen value of *k*, the value of *n* varies among cases and is found with a computer-intensive search algorithm. An example is shown of the two space-time nearest-neighbors of a given index case, by taking *n* = 7 at a radius of 15 km (in space), and 13 days (in time). The order is determined by increasing or decreasing the space-time intersection.

Clustering of meningococcal cases is defined as excess occurrence of the same serosubtype in patients, in spatial and temporal proximity. We used patients' residences as "place" and chose the first day of illness as "time." The actual incidence of clustering was compared to the incidence that would be expected by chance, by using space-time nearest-neighbor analysis (Figure 1). To quantify the phenomenon of clustering, we defined the concept of space-time nearest-neighborship as follows. We defined *nt* nearest-neighbors in time of case 1 as the *n* cases that occur closest (in time) to case 1. Similarly the *np* nearest-neighbors in place of case 1 are the *n* cases that occur closest in space to case 1. The distance between cases is defined as the distance in a straight line between the geographic centers of the reported cases (municipality or postal code area). The *k* cases that are both *nt* nearest-neighbors in time and *np* nearest-neighbors in place (intersection of place and time), are now the group of the 1st, 2nd, …, and *k*th nearest-neighbors (i.e., nearest in both place and time). The order (first, second, and so on) is set in such a way that *k* = 1 defines the first nearest-neighbor, *k* = 2 defines the second nearest-neighbor, and so on. A program was written in C to analyze *k*th nearest-neighborship. This program is available from the authors.

**Figure 2:**
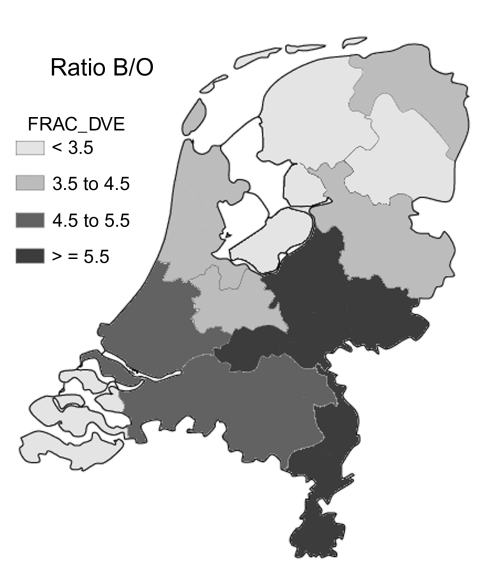
Distribution of the ratio of serogroup B to other serogroups (Ratio B/O) per province in the Netherlands (1993–2001).

First, we calculated the "background" probability that a *k*th nearest-neighbor is of the same strain, under no clustering as the null hypothesis, by calculating the frequency of having a *k*th nearest-neighbor of the same strain when the observed strains are randomly assigned to the observed dates and places of actual cases. This shuffling is called random labeling (*5**,**6*). The null hypothesis assumes complete homogeneity in space and time, which is plausible for small areas within a short time (e.g., 1 year); however, spatial and temporal heterogeneity may give rise to spurious clustering. The prevalence of serogroup B was not constant during the 9 years of our study (Table 1), and the ratio of serogroup B to other serogroups varies somewhat by region (Figure 2). Therefore, this concept of "random labeling" may not apply to our meningococcal data, since it ignores regional differences in occurrence and slow trends in the presence of certain serosubtypes over the period of observation. Thus, random labeling would underestimate the true null (under no clustering) background probability that a nearest-neighbor is of the same strain, thereby overestimating clustering. We decided that the true null background probability is best estimated by the observed frequency of the mean of the 6th to 10th nearest-neighbors (the null probability of no clustering), which assumes clustering is a priori implausible beyond the 5th nearest-neighbor. We calculated 95% confidence intervals (CI) for the excess chance that the first, second, third, fourth, and fifth nearest-neighbor is of the same strain by using paired t-tests. These paired t-tests were carried out on a) the indicator (0/1) variable, indicating whether the first, second, third, fourth, or fifth nearest-neighbor is of the same strain, and b) the average of five such indicator variables for the 6th to 10th nearest-neighbor. The above analyses were calculated for all cases combined but also separately for serogroups B, C, and W135 and for each serosubtype separately.

## Results

During the 9-year surveillance period, 4,896 confirmed cases were noted. Of these, nine cases could not be used because of recording errors (Table 1). The dataset was made up of 250 different meningococcal serosubtypes, of which 42 were seen in 20 or more cases (4,189/4,887 = 86% of all strains), while 99 serosubtypes were only connected to one case (Table A1).

The observed background value of cases in temporal and spatial proximity to an index case being of the same serosubtype is 12.0%. When random labeling was used, this percentage was 9.7%. We observed that 15.1% of the first nearest-neighbors were of the same serosubtype, an excess probability or secondary case percentage of 3.1% (CI 2.1%–4.1%). As most nearest-neighbors are coincidental, little difference was seen in the mean temporal and spatial distance between nearest-neighbors of the same serosubtype (6.1 km [range 0–44 km] and 13.2 days [range 0–63 days]) and those of different serosubtype (7.6 km [range 0–49 km] and 14.3 days [range 0–380 days]). The probability of the second, third, fourth, and fifth nearest-neighbors being of the same serosubtype did not differ significantly from background values (this difference was 0.6%, 0.3%, 0.8%, and 0.4%, respectively). For serogroup B, the excess probability was 3.1% (CI 2.0%–4.3%, n = 4,035) for the first nearest-neighbor. For serogroup C, the excess probability was 3.5% (CI 1.6%–5.3%; n = 728), and for serogroup W135 no excess probability was found (n = 59). Seven different serosubtypes, accounting for 14% (694/4,887) of all cases, showed significant excess probability (Table 2): B:1:P1.4 (12%), B:1:P1.16 (10%), B:4:P1.5 (20%), B:4:P1.10 (5%), B:nt:P1.nt (11%), B:15:P1.7 (11%), and B:15:P1.7,16 (7%).

**Table 2:** Clustering of meningococcal disease cases by serosubtype (serogroup, serotype, and subtype)^a,b^

Serosubtypes (phenotype)	Field clusters^c^	Cases/cluster	% excess probability	95% CI	n
B:1:P1.4	1 Primary school	3	**12.0**	4.2%–19.7%	87
B:1:P1.14	1 Swimming pool	4	7.2	NS (–8.0% to 22.4%)	25
B:1:P1.16	1 Primary school	3	**10.0**	2.4%–17.6%	92
	1 Daycare center	2			
	1 Household	2			
B:4:P1.2,5	1 Household	2	1.9	NS (–3.8% to 7.7%)	52
B:4:P1.4	2 Primary schools	3 and 5	2.0	NS (–0.7% to 4.6%)	1,376
	4 Primary schools	2			
	2 Daycare centers	2 and 3			
	6 Households	2			
B:4:P1.5	1 Daycare center	2	**20.0**	3.2%–36.9%	25
B:4:P1.7	NFC		6.4	NS (–2.7% to 15.5%)	47
B:4:P1.9	1 Primary school	3	7.2	NS (–8.0% to 22.4%)	36
B:4:P1.10	1 Household	2	**4.8**	0.1%–9.5%	205
B:4:P1.14	NFC		5.9	NS (–2.5% to 14.2%)	34
B:4:P1.15	NFC		4.0	NS (–0.4% to 8.4%)	129
B:4:P1.16	1 Household	2	2.2	NS (–2.4% to 6.8%)	63
B:4:P1.NT	1 Primary school	2	2.6	NS (–0.8% to 6.1%)	455
	1 Daycare center	2			
	1 Household	3			
B:NT:P1.14	2 Households	2 and 3	13.0	NS (–1.9% to 27.9%)	23
B:NT:P1.15	1 Household	2	7.2	NS (–1.7% to 16.0%)	39
B:NT:P1.16	1 Household	2	10.0	NS (–4.4% to 24.4%)	20
B:NT:P1.NT	1 Household	2	**10.7**	4.7%–16.8%	123
B:14:P1.4	1 Daycare center	2	0.9	NS (1.4% to 12.2%)	85
	1 Household	2			
B:15:P1.7	NFC		**11.3**	0.1%–22.6%	53
B:15:P1.7,16	1 Primary school	2	**6.8**	1.4%–12.2%	109
B:15:P1.9	1 Household	2	10	NS (–1.4% to 21.4%)	30
B:16:P1.14	1 Household	2	— ^d^		10
B:16:P1.2,5	1 Daycare center	3	13.6	NS (–1.9% to 29.2%)	22
C:2a:P1.2,5	1 Household	2	–1.0	NS (–5.4% to 3.4%)	164
C:NT:P1.5	NFC		10	NS (–4.4% to 24.4%)	20
C:14:P1.12	1 Household	2	— ^d^		2

^a^The following results are shown: serosubtypes with reported field clusters, serosubtypes with significant excess probability for clustering by nearest-neighbor analysis, and serosubtypes with nonsignificant excess probability of more than 3% (**bold** percentages significant). ^b^CI, confidence interval; NT, not typable; NS, not significant. ^c^NFC, no field cluster was reported for this serotype. ^d^Calculating excess probability not possible because n is too small.

The Municipal Public Health Services identified 40 field clusters involving 21 different serosubtypes: 11 primary school clusters (range 2–5 cases), 7 daycare center clusters (2–3 cases), 1 swimming pool cluster (4 cases), and 21 household clusters (2–3 cases). The cases all occurred within 21 days from the first case, and 78% (32/41) occurred within 8 days.

Six serosubtypes were identified by both methods as serosubtypes with clustering, 15 were identified only in field clusters, and 1 in statistical clustering only. Most field clusters consisted of only two cases (75%); this result is consistent with the results of our statistical approach.

## Discussion

Our results suggest that in the context of current public health efforts, clustering of meningococcal disease is rare in the Netherlands and other Western countries. Our nearest-neighbor analysis provided a useful method of assessing the phenomenon of meningococcal clustering by taking random variance into account. Cases of the same serosubtype appeared beyond the expected background rate and were only seen in the first nearest-neighbor, which implies that only secondary cases occur in excess of chance (3.1%). Connections of more than two cases could not be demonstrated beyond chance. Throughout the year, invasive disease appears mostly as isolated cases. This limited clustering may reflect the positive effect of the prophylactic rifampicin policy; however, household field clusters are still reported, which possibly shows the constraints of this prevention policy. This paucity of real secondary cases is consistent with findings from other studies. A Belgian study found 4.4% secondary cases (range 2.0%–5.2%) in 1,913 cases of invasive meningococcal disease from 1971 through 1976 (*7*). In France, 37 (4.5%) co-primary and secondary cases were found in 814 reported cases from 1997 to 1988 (*8*). A Dutch study reported 1.4% co-primary and secondary cases among 507 cases from 1989 to 1990 (*9*). In England and Wales, 17 (0.5%) secondary cases were found among 3,256 cases from 1984 through 1987 (*10*). In a Danish study published in 2000, 1.2% secondary cases were observed in 172 cases of meningococcal disease (*11*).

Apart from proper prophylactic treatment, no additional measures could prevent further cases, since excess clustering only occurs in the first nearest-neighbors, while a cluster is only identified after at least two connected cases. The field cluster analysis confirms this assessment, since most new cases occur within a short period (78% within 8 days), occur geographically close to each other (patients are in the same household, daycare center, or primary school), and occur mostly in pairs (75%). These findings are consistent with observations in field cluster studies showing that secondary invasive disease most likely occurs nearby, within the next few days. In a Belgian study, 83% of 63 secondary cases occurred within 8 days of identifying the index case (*7*); in a French study, 31 (82%) of 38 secondary cases occurred within 8 days (*8*). Almost all (94%, 29/31) of the secondary cases occurred within 8 days in a study in the United States from 1980 to 1993 with eight school and university clusters (*12*). Five secondary cases occurred within 8 days in a school outbreak of six cases with serogroup B meningococcal disease in the United States (*13*).

Space-time clustering methods, e.g., those using the spatial scan-statistic (*14**–**17*), have been used for surveillance purposes with the objective of identifying outbreaks. However, to our knowledge, such methods have not been used to explore the existence of, and quantify, the phenomenon of clustering in a specific infectious disease. For this purpose the Ederer-Myers-Mantel procedure has been used (*18**,**19*); however, since this method requires separating space and time (e.g., into provinces and years), we considered it inappropriate for our purposes. Instead, we adapted the concept of nearest-neighborship to the two dimensions of space and time simultaneously (*5**,**6*).

Our study has several constraints. As many serosubtypes were rare, their individual clustering behavior could not be fully ascertained. We used place of residence as our geographic parameter, which could underestimate clustering, since transmission might occur at locations outside place of residence (such as work, school, and sport clubs). Most cases are found in children, who often spend time in daycare centers, schools, and other places outside the home. Since these places tend to be located in the same area as their homes, this factor likely did not affect our results. The extent of clustering was possibly overestimated because of imprecise geographic coordinates since our statistical method used the center of the municipality or postal code area, but no more precise alternative is available. Since only phenotypic strain typing was conducted (serogroup, serotype, and subtype) and not the more sensitive porA-genotyping method that would have identified spurious clusters, background rates of clustering may have been overestimated. However, this method is unlikely to have affected the excess probability (3.1%) of clustering, since this rate is probably a result of direct transmission. Our method for calculating background value was chosen to be as realistic as possible; however, our results do not appear to be sensitive to the choice of 6th to 10th nearest-neighbors as a reference. For instance, results from 3rd to 10th nearest-neighbor or 7th to 10th nearest-neighbor, as a reference, were virtually identical.

We believe that our low observed incidence of secondary cases partly reflects the general inability to link cases connected by chains of transmission. As disease develops in only a few of the links in a chain of transmission, connected cases are unlikely to be still temporally and spatially close, which obviates detection. Not surprisingly, we found three times as many serosubtypes among reported field clusters (21 serosubtypes) than assessed with nearest-neighborship analysis (7 serosubtypes), which confirms that field clusters may be spurious. Although field clusters have low specificity, their sensitivity is presumably high. Genotyping can identify those clusters brought about by direct transmission; nevertheless, the value of cluster surveillance as a means of prevention is uncertain. Apparent clusters are not valuable to guide additional intervention efforts, since these would prevent few additional cases. Our method of space-time nearest-neighborship analysis provides a sensitive novel approach to the epidemiology of meningococcal disease and possibly even other infectious diseases.

## Appendix

**Table A1:** Number of included meningococcal cases by serosubtype (serogroup, serotype, and subtype)

A:4:P1.NT; n = 1	B:15:P1.NT; n = 33	B:4:P1.7,16; n = 4	C:16:P1.16; n = 1	C:NT:P1.2; n = 1
A:NT:P1.16; n = 4	B:16:P1.1; n = 2	B:4:P1.7,9; n = 1	C:16:P1.2; n = 11	C:NT:P1.2,5; n = 5
B:1:P1.1,7; n = 1	B:16:P1.14; n = 10	B:4:P1.9; n = 36	C:16:P1.2,5; n = 18	C:NT:P1.5; n = 20
B:1:P1.10; n = 1	B:16:P1.16; n = 8	B:4:P1.NT; n = 455	C:16:P1.NT; n = 5	C:NT:P1.5,10; n = 1
B:1:P1.12; n = 2	B:16:P1.2; n = 9	B:4,14:P1.14; n = 2	C:2a:P1.1;n = 1	C:NT:P1.6; n = 7
B:1:P1.14; n = 25	B:16:P1.2,5; n = 22	B:4,14:P1.16; n = 4	C:2a:P1.16; n = 8	C:NT:P1.7; n = 7
B:1:P1.15; n = 2	B:16:P1.2,7; n = 1	B:4,14:P1.4; n = 4	C:2a:P1.2; n = 42	C:NT:P1.7,9; n = 1
B:1:P1.16; n = 92	B:16:P1.4; n = 1	B:4,14:P1.5; n = 1	C:2a:P1.2,5; n = 164	C:NT:P1.9; n = 1
B:1:P1.4; n = 87	B:16:P1.5; n = 3	B:4,14:P1.9; n = 1	C:2a:P1.4; n = 4	C:NT:P1.NT; n = 9
B:1:P1.4,14; n = 1	B:16:P1.6; n = 2	B:4,14:P1.NT; n = 4	C:2a:P1.5; n = 163	W135:2a:P1.2,5; n = 16
B:1:P1.5; n = 15	B:16:P1.7; n = 3	B:4,15:P1.10; n = 1	C:2a:P1.6; n = 2	W135:4,15:P1.16; n = 1
B:1:P1.5,9; n = 1	B:16:P1.7,16; n = 1	B:4,15:P1.4; n = 2	C:2a:P1.7,15; n = 1	W135:NT:P1.1,7; n = 5
B:1:P1.6; n = 3	B:16:P1.NT; n = 9	B:4,15:P1.7; n = 1	C:2a:P1.9; n = 3	W135:NT:P1.2; n = 1
B:1:P1.7,16; n = 7	B:2a:P1.1; n = 1	B:4,15:P1.NT; n = 1	C:2a:P1.NT; n = 44	W135:NT:P1.2,5; n = 2
B:1:P1.9; n = 1	B:2a:P1.16; n = 1	B:4,16:P1.4; n = 1	C:2a,1:P1.16; n = 1	W135:NT:P1.5; n = 5
B:1:P1.9,12; n = 1	B:2a:P1.2; n = 1	B:4,16:P1.7; n = 2	C:2b:P1.1; n = 1	W135:NT:P1.6; n = 25
B:1:P1.NT; n = 61	B:2a:P1.2,5; n = 2	B:NT:P1.1; n = 3	C:2b:P1.1,7; n = 3	W135:NT:P1.NT; n = 4
B:14:P1.1; n = 2	B:2a:P1.4,5; n = 1	B:NT:P1.1,7; n = 10	C:2b:P1.10; n = 9	X:4:P1.16; n = 3
B:14:P1.1,7; n = 1	B:2a:P1.7,16; n = 1	B:NT:P1.10; n = 23	C:2b:P1.14; n = 2	X:4:P1.7; n = 1
B:14:P1.12; n = 9	B:2a:P1.NT; n = 3	B:NT:P1.10,14; n = 1	C:2b:P1.15; n = 1	X:4:P1.7,15; n = 1
B:14:P1.14; n = 2	B:2a,1:P1.2,5; n = 1	B:NT:P1.12; n = 9	C:2b:P1.16; n = 1	X:4:P1.NT; n = 1
B:14:P1.15; n = 17	B:2a,1:P1.NT; n = 1	B:NT:P1.14; n = 23	C:2b:P1.2; n = 5	X:NT:P1.16; n = 1
B:14:P1.16; n = 1	B:2b:P1.1,7; n = 1	B:NT:P1.15; n = 39	C:2b:P1.2,12; n = 2	Y:14:P1.14; n = 1
B:14:P1.2,5; n = 3	B:2b:P1.10; n = 34	B:NT:P1.16; n = 20	C:2b:P1.2,5; n = 66	Y:14:P1.2,5; n = 2
B:14:P1.4; n = 85	B:2b:P1.12; n = 1	B:NT:P1.2; n = 5	C:2b:P1.4; n = 1	Y:14:P1.5; n = 1
B:14:P1.4,15; n = 1	B:2b:P1.15; n = 1	B:NT:P1.2,5; n = 20	C:2b:P1.5; n = 2	Y:14:P1.7,14; n = 1
B:14:P1.4,7; n = 1	B:2b:P1.2,5; n = 8	B:NT:P1.2,7; n = 1	C:2b:P1.NT; n = 8	Y:14:P1.NT; n = 4
B:14:P1.5; n = 4	B:2b:P1.7; n = 1	B:NT:P1.4; n = 102	C:4:P1.1; n = 16	Y:15:P1.16; n = 1
B:14:P1.5,10; n = 1	B:2b:P1.NT; n = 9	B:NT:P1.4,12; n = 2	C:4:P1.1,7; n = 1	Y:15:P1.5; n = 1
B:14:P1.6; n = 1	B:4:P1.1; n = 4	B:NT:P1.4,7; n = 2	C:4:P1.10; n = 5	Y:4:P1.5; n = 7
B:14:P1.7; n = 1	B:4:P1.1,7; n = 15	B:NT:P1.5; n = 8	C:4:P1.14; n = 1	Y:4:P1.NT; n = 1
B:14:P1.7,16; n = 1	B:4:P1.10; n = 205	B:NT:P1.6; n = 30	C:4:P1.15; n = 4	Y:4,14:P1.5; n = 3
B:14:P1.9; n = 3	B:4:P1.12; n = 12	B:NT:P1.7; n = 21	C:4:P1.16; n = 1	Y:NT:P1.12; n = 1
B:14:P1.NT; n = 36	B:4:P1.12,16; n = 1	B:NT:P1.7,16; n = 3	C:4:P1.2,5; n = 2	Y:NT:P1.14; n = 1
B:15:P1.1,7; n = 2	B:4:P1.14; n = 34	B:NT:P1.9; n = 42	C:4:P1.4; n = 13	Y:NT:P1.15; n = 11
B:15:P1.10; n = 14	B:4:P1.14,15; n = 4	B:NT:P1.NT; n = 123	C:4:P1.4,15; n = 1	Y:NT:P1.6; n = 2
B:15:P1.12; n = 1	B:4:P1.15; n = 129	C:1:P1.1; n = 2	C:4:P1.4,7; n = 1	Y:NT:P1.7; n = 1
B:15:P1.14; n = 2	B:4:P1.16; n = 63	C:1:P1.16; n = 4	C:4:P1.5; n = 2	Z:NT:P1.16; n = 2
B:15:P1.15; n = 11	B:4:P1.2; n = 9	C:1:P1.5; n = 1	C:4:P1.6; n = 1	Z:NT:P1.NT; n = 1
B:15:P1.16; n = 27	B:4:P1.2,5; n = 52	C:1:P1.7; n = 1	C:4:P1.7; n = 1	Ng:14:P1.2,5; n = 1
B:15:P1.2,5; n = 5	B:4:P1.2,7; n = 1	C:1:P1.NT; n = 7	C:4:P1.9; n = 2	Ng:14:P1.NT; n = 1
B:15:P1.4; n = 77	B:4:P1.4; n = 1376	C:14:P1.12; n = 2	C:4:P1.NT; n = 6	Ng:15:P1.7,16; n = 1
B:15:P1.4,7; n = 2	B:4:P1.4,15; n = 4	C:14:P1.2,5; n = 3	C:NT:P1.1; n = 3	Ng:2b:P1.10; n = 1
B:15:P1.5; n = 12	B:4:P1.4,7; n = 17	C:14:P1.4; n = 1	C:NT:P1.1,7; n = 4	Ng:4:P1.10; n = 1
B:15:P1.6; n = 8	B:4:P1.5; n = 25	C:14:P1.7,16; n = 1	C:NT:P1.1,7; n = 4	Ng:4:P1.4; n = 1
B:15:P1.7; n = 53	B:4:P1.6; n = 29	C:14:P1.NT; n = 1	C:NT:P1.10; n = 4	Ng:NT:P1.1,7; n = 1
B:15:P1.7,14; n = 1	B:4:P1.7; n = 47	C:15:P1.7; n = 1	C:NT:P1.12; n = 1	Ng:NT:P1.2,5; n = 1
B:15:P1.7,15; n = 2	B:4:P1.7,10; n = 2	C:15:P1.7,16; n = 2	C:NT:P1.14; n = 1	29^E^16:P1.2,5; n = 2
B:15:P1.7,16; n = 109	B:4:P1.7,14; n = 1	C:15:P1.NT; n = 1	C:NT:P1.15; n = 1	29^E^NT:P1.2,5; n = 1
B:15:P1.9; n = 30	B:4:P1.7,15; n = 2	C:16:P1.1,7; n = 4	C:NT:P1.16; n = 1	29^E^NT:P1.NT; n = 1
